# Fluorescent indolizine derivative YI-13 detects amyloid-β monomers, dimers, and plaques in the brain of 5XFAD Alzheimer transgenic mouse model

**DOI:** 10.1371/journal.pone.0243041

**Published:** 2020-12-23

**Authors:** DaWon Kim, Jeong Hwa Lee, Hye Yun Kim, Jisu Shin, Kyeonghwan Kim, Sejin Lee, Jinwoo Park, JinIkyon Kim, YoungSoo Kim

**Affiliations:** 1 Department of Pharmacy and Yonsei Institute of Pharmaceutical Science, College of Pharmacy, Yonsei University, Incheon, Republic of Korea; 2 BioActs, Incheon, Republic of Korea; Nathan S Kline Institute, UNITED STATES

## Abstract

Alzheimer disease (AD) is a neurodegenerative disorder characterized by the aberrant production and accumulation of amyloid-β (Aβ) peptides in the brain. Accumulated Aβ in soluble oligomer and insoluble plaque forms are considered to be a pathological culprit and biomarker of the disorder. Here, we report a fluorescent universal Aβ-indicator **YI-13**, 5-(4-fluorobenzoyl)-7,8-dihydropyrrolo[1,2-*b*]isoquinolin-9(6*H*)-one, which detects Aβ monomers, dimers, and plaques. We synthesized a library of 26 fluorescence chemicals with the indolizine core and screen them through a series of *in vitro* tests utilizing Aβ as a target and **YI-13** was selected as the final imaging candidate. **YI-13** was found to stain and visualize insoluble Aβ plaques in the brain tissue, of a transgenic mouse model with five familial AD mutations (5XFAD), by a histochemical approach and to label soluble Aβ oligomers within brain lysates of the mouse model under a fluorescence plate reader. Among oligomers aggregated from monomers and synthetic dimers from chemically conjugated monomers, **YI-13** preferred the dimeric Aβ.

## Introduction

Alzheimer disease (AD) is the most common type of dementia with unique accumulation of misfolded amyloid-β (Aβ) peptides in the brain [1]. Alzheimer brains show an increased Aβ production and aggregation in addition to a decreased Aβ clearance and degradation, leading to the neurodegeneration. Since the early stage of AD, highly aggregation-prone soluble Aβ monomers form soluble oligomers and insoluble plaques in hippocampus and cortex, which regulate learning and memory abilities [2, 3]. Aβ plaques are found less associated with neurodegeneration and clinical severity of AD, given that they are often deposited at a distance sites of neuronal loss and their clearance barely ameliorated cognitive impairments [4, 5]. Instead, soluble Aβ oligomers were found to play an important role in the pathogenesis of AD as they exhibited potent neurotoxicity with higher correlation with AD severity [6–9]. Current brain imaging for AD diagnosis, however, is mostly limited to targeting insoluble forms of Aβ by lack of detection methods of monomers and oligomers and, thus, alterations of soluble Aβ species in the brain cannot be utilized as end points of drug clinical trials [10]. Given that soluble Aβ monomers and oligomers are not identical to insoluble plaques in their pathogenic roles, detection of all species of Aβ, soluble and insoluble forms, in the brain may provide additional information for the better differential diagnosis of AD.

**Fig 1:**
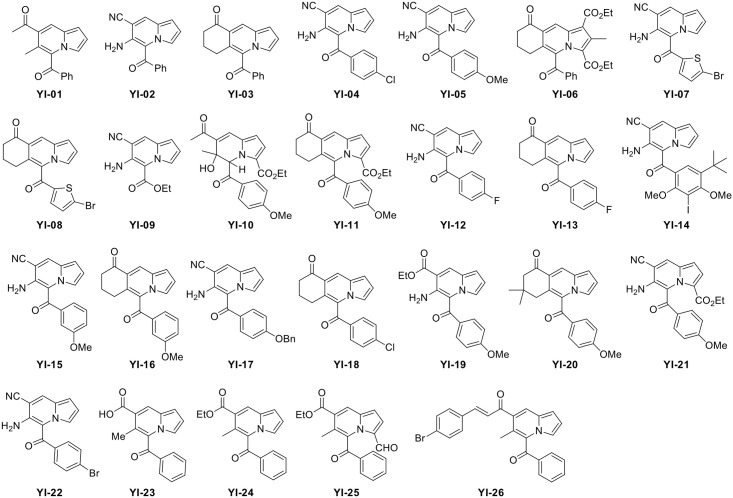
Chemical structures of YI compounds. A chemical library of 26 fluorescent indolizine derivatives.

Here, we prepared a chemical library of indolizine derivatives targeting Aβ (Fig 1). The library was initially aimed to find compounds which can inhibit Aβ aggregation and/or dissociate pre-formed Aβ fibrils, while none of them has affected Aβ deposition. Instead, we serendipitously found that several compounds showed changes in fluorescent intensities in the presence of either Aβ monomers or aggregates, in which 5-(4-fluorobenzoyl)-7,8-dihydropyrrolo[1,2-*b*]isoquinolin-9(6*H*)-one (**YI-13**) showed the highest increase upon interactions. **YI-13** was further examined as an imaging probe targeting monomeric and aggregated Aβ by *in vitro* and *ex vivo* analyses using synthetic Aβ and 5XFAD transgenic AD mouse model, respectively. Upon sacrifice and brain extraction of aged and young 5XFAD mice, we prepared two different brain samples; a half brain of sliced frozen sections and the other half for lysates. **YI-13** was applied to brain slides for the visualization of insoluble plaque in histochemical analyses and to brain lysates for the detection of soluble oligomers in fluorescence spectrum reading. In order to investigate specific targeted forms among heterogenous soluble oligomers, **YI-13** was employed to low-molecular weight oligomeric Aβ isolated by the size cut-off filtration and dimeric Aβ synthesized by C-terminal conjugation, and fluorescence emission spectrum were compared.

## Results

### Aβ monomers and aggregates increase fluorescent intensity of YI compounds

In this study, we synthesized 26 indolizine-derived **YI** compounds as a chemical library targeting Aβ. Among them, 23 **YI** compounds were previously reported chemicals and **YI-09**, **YI-10**, and **YI-23** were newly designed [11]. Indolizine, an isomer of indole, consists of 5- and 6-membered heterocyclic rings with a nitrogen atom [12]. Since compounds containing indolizine moiety possess a broad spectrum of valuable pharmacological activities such as anti-inflammatory, antimicrobial, anticancer, and antioxidant properties, various strategies for generating novel collections with indolizine scaffold have been developed [13, 14]. Despite the several useful activities for AD, such as anti-inflammatory and oxidant properties, specific application of the indolizine scaffold to AD has not been reported yet.

**Fig 2:**
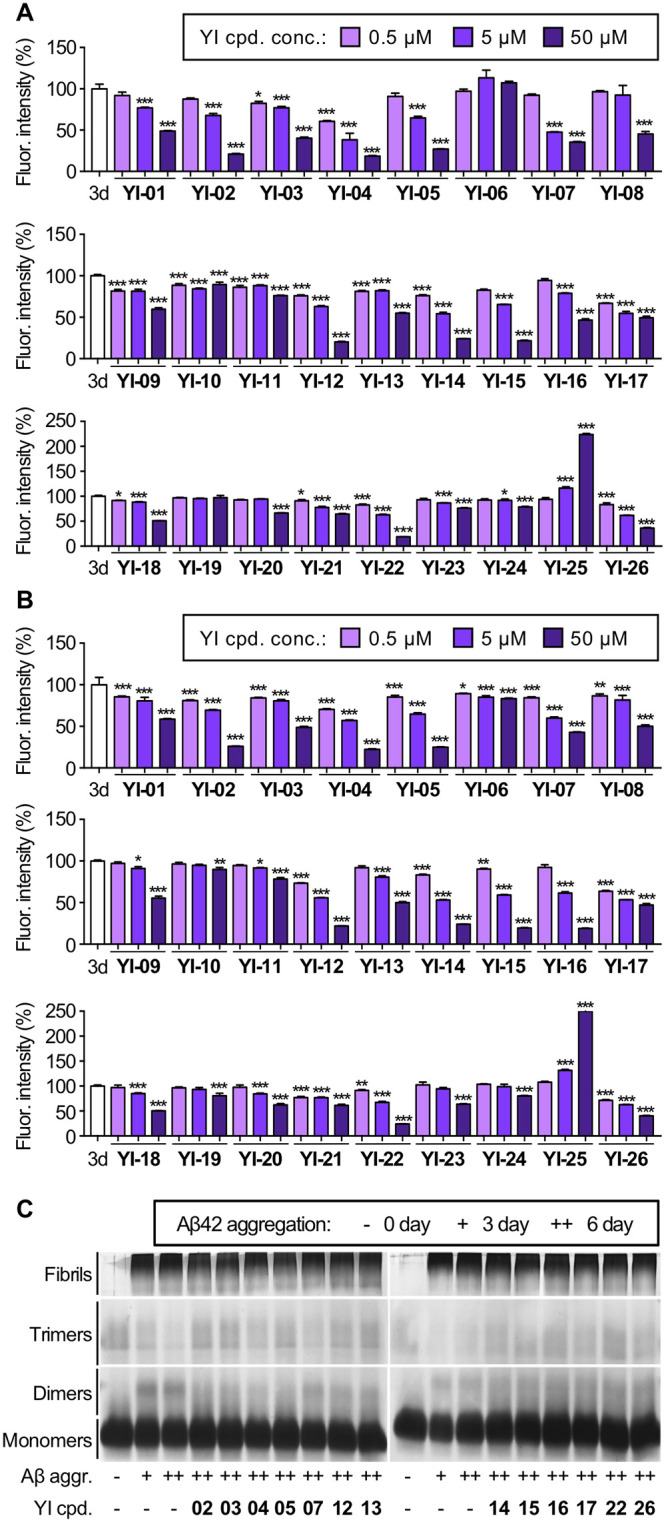
*In vitro* assays to evaluate anti-amyloidogenic properties of indolizine-derived YI compounds. (A) Aβ aggregation inhibition test by ThT assay. Aβ42 (50 μM) was incubated with or without **YI** compounds (0.5, 5, 50 μM) for three days. (B)Aβ aggregates dissociation test by ThT assay. Aβ42 aggregates (50 μM, 3-day pre-aggregation) were incubated with or without **YI** compounds (0.5, 5, 50 μM) for additional three days. (C) SDS-PAGE with PICUP and silver staining for disaggregated Aβ (50 μM) with the incubation of YI compounds (250 μM). Sizes of Aβ species according to the protein size markers are monomers (5 kDa), dimers (10 kDa), oligomers (15 to 75 kDa), and larger aggregates or fibrils (embedded at the top of the gels). Fluorescent intensities of all samples were normalized to 3-day Aβ aggregates data (100%). Denormalized data of ThT assay (S1 Fig) and whole gel images are shown in the (S2 Fig). Abbreviations: 3d = 3-day incubation of Aβ,– = Aβ monomer, + = 3-day incubation of Aβ, ++ = 3-day pre-incubation of Aβ and additional 3-day incubation of Aβ and/or compounds. Data represents the mean of triplicated experiments ± SEMs and one-way anova was applied followed by Bonferroni’s post-hoc comparison test (*P < 0.033, **P < 0.002, ***P < 0.001).

The primary screening of the compounds utilized thioflavin T (ThT), a commonly used chemical reagent that exhibit red-shifted fluorescence upon binding to β-sheet-rich protein complex [15]. Through ThT assays, effects of total **YI** compounds in either inhibiting the Aβ aggregation or dissociating pre-formed Aβ aggregates were examined. In Aβ aggregation inhibition assay, we tested three concentrations of all **YI** compounds, 0.5, 5, and 50 μM. Each **YI** compound was added to monomeric Aβ42 (50 μM) before the incubation for fibril formation. Among the total compounds, 14 compounds, **YI-01**, **YI-02**, **YI-03**, **YI-04**, **YI-05**, **YI-07**, **YI-08**, **YI-12**, **YI-14**, **YI-15**, **YI-16**, **YI-17**, **YI-22**, and **YI-26**, were found to have significant inhibitory effects on Aβ fibril formation by showing 50% or less fluorescent intensity of ThT compared to the control in chemical concentration of 50 μM (Fig 2A). Next, we performed the Aβ-aggregates dissociation assay on the same library with three concentrations, 0.5, 5, 50 μM. We incubated Aβ42 (50 μM) solely for three days to obtain pre-formed aggregates. Pre-formed Aβ42 was then mixed with each compound to induce disaggregation. In similar fashion to the inhibition assay, 13 compounds, **YI-02**, **YI-03**, **YI-04**, **YI-05**, **YI-07**, **YI-12**, **YI-13**, **YI-14**, **YI-15**, **YI-16**, **YI-17**, **YI-22**, and **YI-26**, were found to reduce Aβ42 fibrils by showing 50% or less fluorescent intensity of ThT compared to the control in chemical concentration of 50 μM (Fig 2B). Besides, Aβ42 treated with **YI-25** showed higher fluorescent intensity than the control in both inhibition and dissociation assays, possibly because **YI-25** accelerates the Aβ aggregation.

Through both ThT fluorescence assays, 14 **YI** compounds were selected for their significant inhibitory activities on Aβ aggregation, and 13 **YI** compounds were selected for their substantial disaggregating activities on pre-formed aggregates, with 12 compounds overlapping. To validate the effectiveness of the selected compounds on Aβ oligomers and protofibrils which consist of immature β-sheets, we employed sodium dodecyl sulfate polyacrylamide gel electrophoresis (SDS-PAGE) analysis for further investigation. Since the folded structure of Aβ aggregates will be denatured in a detergent environment such as SDS-PAGE, photo-induced cross-linking of unmodified proteins (PICUP) was initially conducted to secure original states of Aβ aggregates, preventing any dissociation [16]. The electrophoretic analysis showed that none of the selected compounds (250 μM) have dissociated Aβ aggregates (50 μM), in comparison to Aβ only controls (Fig 2C; full-length gel images are available in S2 Fig). This result is conflicted with the data from ThT assays and indicates that anti-amyloidogenic activities of **YI** compounds might be false-positive results.

**Fig 3:**
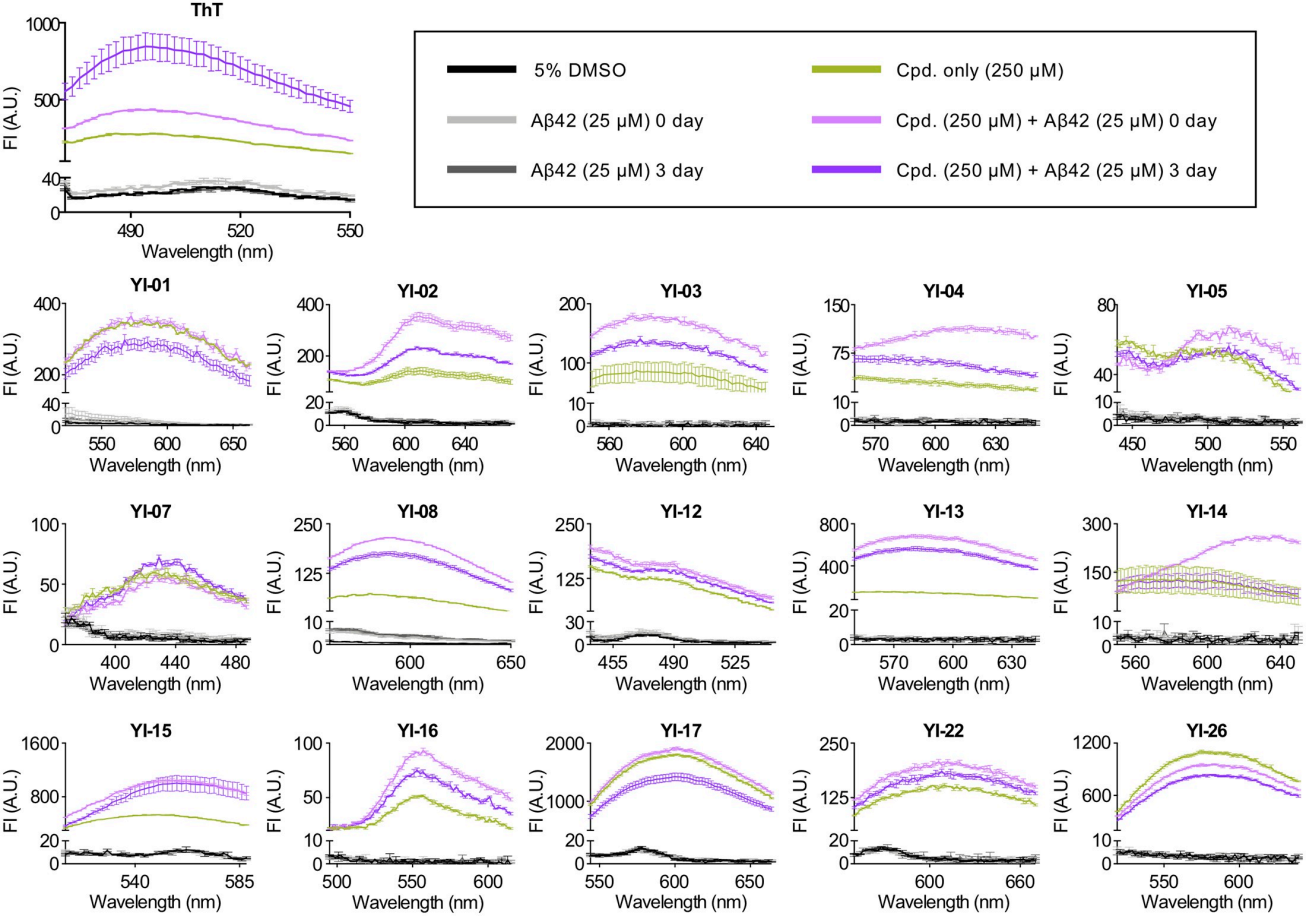
Fluorescence spectra of YI compounds in the presence of Aβ42. Selected 15 **YI** compounds (250 μM) were added to 25 μM Aβ42 monomers or aggregates obtained by 3-day incubation. Emission spectra of **YI** compounds with Aβ42 in 3:1 ratio were recorded with the individual excitation wavelength as following: **YI-01**, 362 nm; **YI-02**, 472 nm; **YI-03**, 330 nm; **YI-04**, 412 nm; **YI-05**, 320 nm; **YI-07**, 332 nm; **YI-08**, 310 nm; **YI-12**, 410 nm; **YI-13**, 394 nm; **YI-14**, 440 nm; **YI-15**, 475 nm; **YI-16**, 330 nm; **YI-17**, 486 nm; **YI-22**, 480 nm; **YI-26**, 415 nm. Fluorescent intensity of ThT (5 μM) in the presence of Aβ42 monomers or aggregates (25 μM) was measured as a control. Spectra of all samples were acquired using an Infinite 200 PRO plate reader. Abbreviations: Cpd. = compound, FI = fluorescence intensity, AU = arbitrary unit.

Due to this discrepancy between the ThT assays and electrophoresis, we presumed that **YI** compounds may have interfered with the ThT and decreased ThT fluorescent intensity, leading to the false-positive results in the screening. There is a possibility that total 15 compounds selected from the ThT assays can significantly bias fibril-associated ThT fluorescence by directly interacting or competitively binding with ThT [17]. To investigate the fluorescent properties of selected compounds, **YI-01**, **YI-02**, **YI-03**, **YI-04**, **YI-05**, **YI-07**, **YI-08**, **YI-12**, **YI-13**, **YI-14**, **YI-15**, **YI-16**, **YI-17**, **YI-22**, and **YI-26** (250 μM) were verified via fluorescence spectral scan. First, the absorbance spectrum of each compound was performed to decide the excitation wavelength which shows up as the highest peak on the spectrum (S3A Fig). Then, the emission spectrum of each compound was achieved using the individual excitation wavelength obtained from the absorbance scanning (S3B Fig). Compounds **YI-01**, **YI-03**, **YI-04**, **YI-05**, **YI-08**, **YI-12**, **YI-13**, **YI-14**, **YI-16**, and **YI-26** exhibited the overlapped wavelengths with the fluorescence spectra of ThT, indicating the possible interferences of **YI** compounds to the ThT assays. Otherwise, **YI** compounds may either interact directly with ThT molecule, decreasing its fluorescent intensity, or competitively bind to β-sheet-rich sites, displacing ThT [18]. These possibilities suggest that **YI** compounds could be utilized as fluorescent probes targeting Aβ. To investigate **YI** compounds as imaging probes targeting Aβ, the selected 15 compounds (250 μM) were added to Aβ monomer (0-day incubation, 25 μM) or aggregates (3-day incubation, 25 μM), then the fluorescent alteration of each compound was measured. Among them, **YI-08**, **YI-13**, and **YI-15** were found to increase fluorescent intensity when mixed with Aβ monomers or aggregates (Fig 3). Compared to the native fluorescent intensity of each **YI** compound itself, **YI-08** showed 212.37% increase in the presence of Aβ monomers and 153.13% increase in the presence of Aβ aggregates; **YI-13** showed 375.69% increase in the presence of Aβ monomers and 289.01% increase in the presence of Aβ aggregates; **YI-15** showed 98.3% increase in the presence of Aβ monomers and 88.41% increase in the presence of Aβ aggregates. We selected **YI-13** for the further animal studies since it exhibited the highest enhancement in fluorescent intensity upon Aβ binding.

### YI-13 detects insoluble Aβ plaques on brain tissues

**Fig 4:**
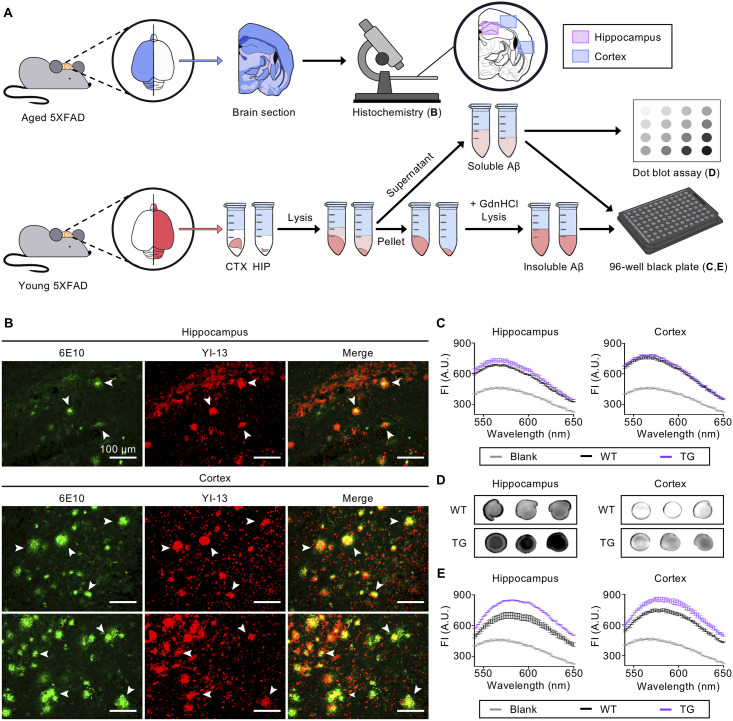
*Ex vivo* analyses of fluorescent YI-13 to confirm it as an imaging agent targeting insoluble and soluble Aβ. (A) A scheme of YI-13 *ex vivo* analyses. A brain hemisphere of aged mice was used for histochemistry (TG and WT, 12-month old, n = 2 and 2). Dissected regions of young mouse brain hemisphere (TG and WT, 5-month old, n = 3 and 3) were lysed to obtain soluble Aβ and verified by fluorescence scans and dot blots. The pellet fraction was further lysed in guanidine-hydrochloride buffer (GdnHCl) to obtain insoluble Aβ and verified by fluorescence scans. The illustration was drawn using Adobe Photoshop software. (B) Brain slides of TG co-stained by 6E10 and **YI-13** (500 μM). Arrowheads indicate Aβ plaques (scale bars = 100 μm). (C-D) Soluble Aβ obtained by brain lysis of TG or WT mouse was analyzed. (C) Fluorescence spectral scan of **YI-13** (250 μM) analyses (394/582 nm, ex/em). (D) Dot blot analyses. The levels of soluble Aβ were determined by using anti-Aβ antibody, 6E10. Original images of blotting analysis are shown in S5 Fig. (E) Insoluble Aβ obtained from pellet lysates was analyzed by fluorescence spectral scan of **YI-13** (250 μM) (394/582 nm, ex/em). Blank in each scanning graph indicates **YI** compound only without any Aβ sample. The data collected from WT littermates is shown in the (S6 Fig). Abbreviations: HIP = hippocampus, CTX = cortex, WT = wild-type, TG = transgenic, AU = arbitrary unit.

A transgenic mouse model co-expressing five FAD mutations (5XFAD) recapitulates major pathologic features of AD with intraneuronal Aβ aggregates formation [19], and the insoluble Aβ plaques in the brain can be monitored using Aβ-specific antibody 6E10 through the immunohistochemical analysis [20, 21]. To confirm the prospects of **YI-13** as an amyloid imaging agent, it was co-stained with 6E10 on the fixed mouse brain tissues of 12-month-old male 5XFAD mouse (Fig 4A). Histochemistry verified that **YI-13** localized on the same sites as 6E10 did on plaques in both the hippocampal and cortical regions (Fig 4B). The overlapped proportion was calculated by dividing number of merged sites with the total number of plaques, both obtained by Image-J software. In particular, 62% of 6E10-stained Aβ plaques in hippocampus and 79% of them in cortex overlapped with **YI-13**. Since wavelength range of **YI-13** overlaps that of secondary antibody conjugated with 6E10, it may raise a concern that **YI-13** staining interfered with 6E10 binding to Aβ aggregates. However, separate histochemical analyses of 6E10 and **YI-13** each on two consecutive brain tissue sections revealed that they both stained the Aβ plaques (S4 Fig). The high fluorescent intensity and precise overlapping of **YI-13** with 6E10 positively suggest it as a promising imaging agent of Aβ plaques.

### YI-13 detects soluble and insoluble Aβ in brain lysates

The formation of soluble Aβ oligomers within the brain is widely accepted to be a main component of AD pathogenesis [22]. To investigate whether **YI-13** can be used as an imaging agent to target oligomeric Aβ, we observed the change in fluorescent intensity of the dye upon mixture with soluble fraction lysates from a 5-month-old female 5XFAD mouse and the age-matched female wildtype mouse (Fig 4A). In order to extract soluble brain lysates, the hippocampal and cortical regions were dissected separately from the mouse brains and homogenized in RIPA buffer. After the centrifugation of the samples, the supernatant, which is the soluble fraction of brain lysates and presumably contains a mixture of monomeric and oligomeric Aβ, was collected. In the hippocampal lysates, **YI-13** displayed 6.65% higher fluorescent intensity in transgenic mice in comparison to wild type mice (Fig 4C). On the other hand, **YI-13** expressed less significant difference in fluorescent intensity when mixed with cortical lysates with only 4.01% increase in transgenic mice compared to wild type mice. These results may be due to higher soluble amyloid concentrations in the hippocampus in comparison to cortical tissues. Previous studies have corroborated that Aβ levels in PDAPP transgenic mice increase age-dependently in the hippocampus and cortex, with the highest expression in the hippocampus [7, 23]. The dot blot assay we performed also shows a significant increase in total Aβ levels in the hippocampus compared to the cortex of the 5XFAD transgenic mouse model (Fig 4D; full-length blot images are available in S5 Fig). Our results suggest that fluorescent **YI-13** is able to target soluble Aβ monomers and oligomers *ex vivo*.

The presence of senile plaques composed of detergent-insoluble Aβ is also observed in AD [24]. To further investigate whether **YI-13** interacts with insoluble Aβ plaques, we prepared detergent-insoluble brain lysates by solubilizing Aβ from plaques with guanidine hydrochloride (GdnHCl) (Fig 4A). GdnHCl extractions, supposedly insoluble fractions, were collected after three hours of shaking followed by two hours of centrifugation. When **YI-13** was added to the insoluble fractions from hippocampal region, its fluorescent intensity increased 21.75% in transgenic mouse models in comparison to wildtype littermates (Fig 4E). Insoluble fractions from cortical region, also, showed significant enhancement in **YI-13** fluorescent intensity with 15.17% increase in transgenic mouse models. **YI-13** could not only target soluble Aβ fractions but also insoluble Aβ lysates.

### YI-13 detects Aβ dimers among oligomers

**Fig 5:**
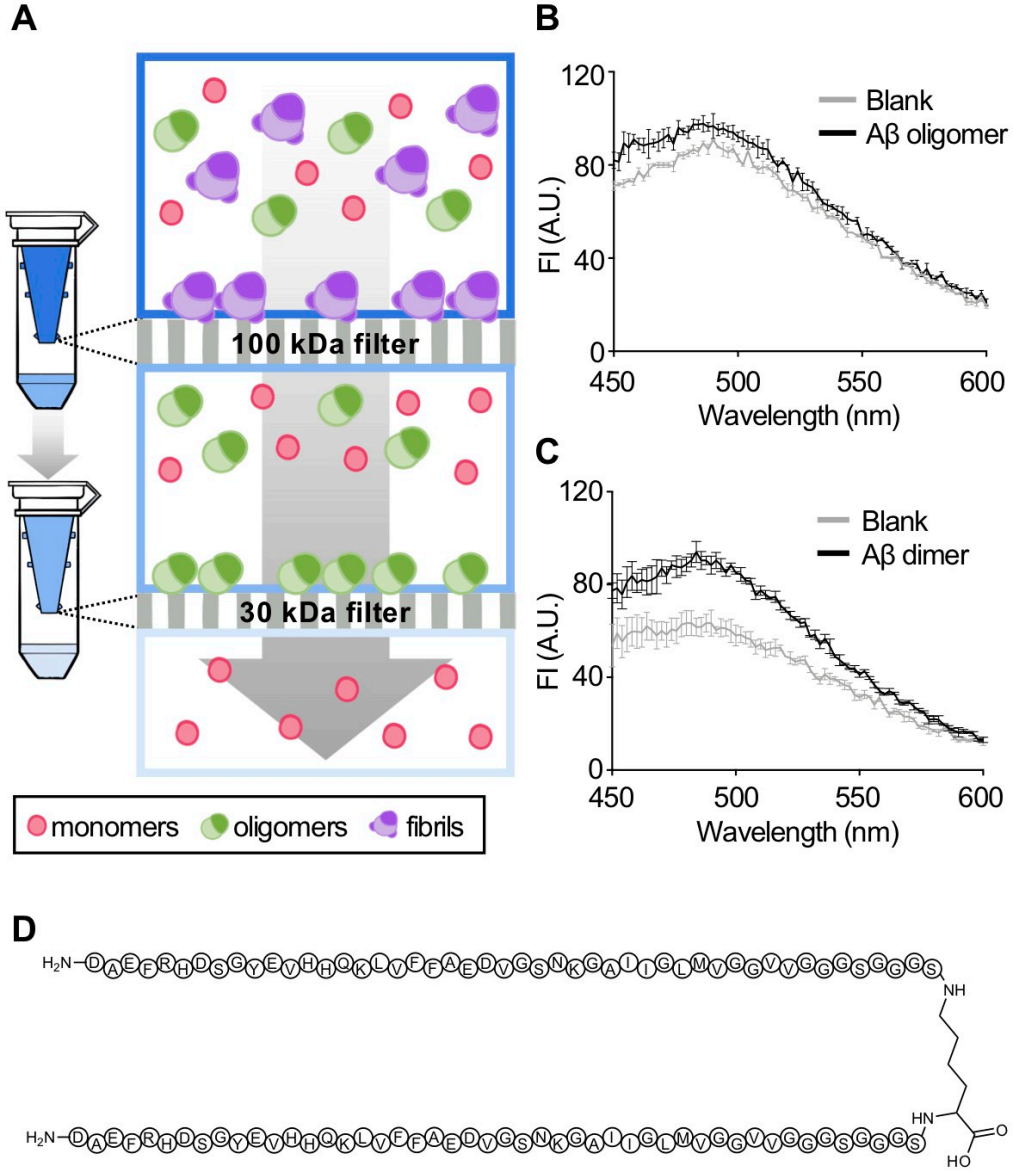
*In vitro* analysis of fluorescent YI-13 to detect its interaction with Aβ oligomers and dimers. (A) A scheme of isolating oligomers from heterogeneous mixture of Aβ aggregates (50 μM). Through 100 kDa membrane filter, high molecular weight Aβ oligomers over 100 kDa were removed from the mixture of Aβ aggregates. The filtrate sample was applied to the 30 kDa filter to exclude low molecular weight Aβ aggregates. Only Aβ oligomers sized from 30 to 100 kDa were used for further experiment. The illustration was drawn using Adobe Photoshop software. (B-C) Fluorescence spectral scan of **YI-13** (250 μM) when applied to Aβ. Blank indicates fluorescent intensities of **YI-13** without Aβ. (B) Fluorescent analyses of **YI-13** with the isolated Aβ oligomers. (C) Fluorescent analyses of **YI-13** with the parallel Aβ dimer (25 μM). (D) Structure of synthesized parallel Aβ dimer with a lysine linker and spacer sequences, (GGGS)_2_. Abbreviations: FI = fluorescence intensity, AU = arbitrary unit.

The plaque staining ability of **YI-13** was validated by colocalization with anti-Aβ monoclonal antibody 6E10 on brain tissues and its monomer detecting function was confirmed by enhanced fluorescent intensity when mixed with monomeric Aβ. However, it is unclear whether the fluorescent signal of **YI-13** was increased by monomers, oligomers, or both in the soluble fraction of 5XFAD brain lysates. Thus, we assessed fluorescent property alteration tests of **YI-13** on isolated oligomers and synthetic full-length dimers of Aβ. Primarily, we isolated oligomers from monomers and fibrils in the heterogeneous mixture of Aβ aggregates by molecular weight cut-off (MWCO) membrane filters of 30 and 100 kDa (Fig 5A). When **YI-13** was applied to the oligomers, only 9.32% increase in fluorescent intensity was observed compared to blank where any kinds of aggregates are excluded. Next, we synthesized a full-length Aβ40 dimer conjugating two C-terminal ends by amide bonds on amines of lysine linker, with flexible spacers (GGGS)_2_, as previously reported, and examined fluorescent property alteration tests of **YI-13** upon the interaction with Aβ dimers [25] (Fig 5C and 5D). Unlike Aβ oligomers, dimers showed a major escalation of 52.43% increase in fluorescent intensity when **YI-13** was added to them. Following results indicate that **YI-13** binds with not only monomers or fibrils but also with oligomers, particularly when they are in dimeric form.

### Fluorescence properties of fluorescent YI-13

The fluorescence characteristics of the **YI-13** were evaluated to determine its extinction coefficient, quantum yield, and brightness. Their alterations in the presence of Aβ monomers (0d) or aggregates incubated for three days (3d) were also measured to compare the parameter changes when **YI-13** is added to Aβ in any forms (Table 1). Compared to the products measured only with **YI-13** solution, the presence of Aβ both in monomeric or fibrillar form led to the decline in all three properties, including extinction coefficient, quantum yield, and brightness. Emission maximum of **YI-13** also showed minor shift in parameters when Aβ, either monomers or aggregates, were added. Considering that **YI-13** indicated monomeric and fibrillar Aβ *in vitro*, stained plaques in the brain tissue, and detected oligomers in the brain lysate despite overall fluorescence properties of the compound became weaker upon interaction with Aβ, the molecular mechanisms behind the interaction of **YI-13** and Aβ needs further investigations.

**Table 1:** Fluorescent properties of selected YI-13 with and without Aβ aggregates.

	Excitation maximum (nm)	Emission maximum (nm)	Extinction (M^-^¹cm^-^¹)	Quantum yield	Brightness
**YI-13**	394	582	6655	0.0011	7.59
**YI-13**+Aβ (0d)	394	586	4065	0.000727	2.95
**YI-13**+Aβ (3d)	394	576	4236	0.000638	2.70

The values of excitation/emission maximum, extinction coefficient, quantum yield, and brightness were measured for **YI-13** itself (500 μM) or when it was added to Aβ monomers (0d, 25 μM) or aggregates (3d, 25 μM).

## Discussion

In this study, we report an indolizine derivative, **YI-13**, a potential imaging probe targeting Aβ. While screening 26 indolizine derivatives for their Aβ-regulating function, we found that the false positive data were obtained due to the possible interferences caused by the presence of exogenous compounds in ThT fluorescence [17] and, instead, we serendipitously discovered that fluorescent intensities of 15 compounds were increased when exposed to Aβ, showing the possibilities as Aβ-imaging probes. Among them, **YI-13** efficiently detected Aβ monomers and aggregates including dimers and plaques. In order to validate the functions of **YI-13**, our primary concern was to obtain and identify its target protein, Aβ, which exists in multiple species. We prepared various forms of Aβ, monomers, dimers, oligomers, fibrils, and plaques, and they were applied for verification of **YI-13** as an Aβ imaging probe. First, we prepared AD mouse brain slides and lysates for detections of plaques and soluble/insoluble Aβ species, respectively. Second, low molecular weight soluble Aβ species were isolated by size cut-off filtrations of heterogenous Aβ aggregates. Additionally, in-house synthetic Aβ parallel dimers were employed to determine the binding ability of **YI-13** to smallest form of Aβ oligomers.

Dimer-preferred binding ability of **YI-13**, which also stains plaques, suggests how this compound interacts with Aβ aggregates. In contrast to common Aβ imaging probes such as ThT and Pittsburgh Compound B (PiB, N-Methyl-^11^C-2-(4’-methylamino-phenyl)-6-hydroxy-benzothiazole) with planar chemical structures to intercalate in β-sheet of insoluble protein aggregates, **YI-13** detects non-β-sheet aggregates and it does not compete with ThT upon fibril binding [26, 27]. **YI-13** targets multiple analytes. One possible interpretation is that **YI-13** targets Aβ dimers and, if the dimeric Aβ conformation is exposed, the larger aggregates including oligomers and plaques can recruit **YI-13**. Immunohistochemical observation revealed that **YI-13** has nonspecific bindings to others, possibly biomolecules, cells, and organelles, on brain tissues beside plaques, and it might be related to the dimer-preferred binding function of the compound. We are looking for methods to investigate the Aβ -binding mechanism and target profiles in the brain. Instead of lacking specificity to a certain form of Aβ species, **YI-13** may detect the change of cerebral Aβ in aggregation and concentration in the earlier stage that plaque-specific probes as it is one a few imaging agents reported to target dimeric Aβ and Aβ oligomer detecting probes are barely developed until the present [10]. Given that **YI-13** already bears fluorine in the chemical structure, its transition to a ^18^F-labeled radiotracer for positron emission tomography could be relatively easy and practical.

## Materials and methods

### Chemical syntheses of indolizine derivatives

**Fig 6:**
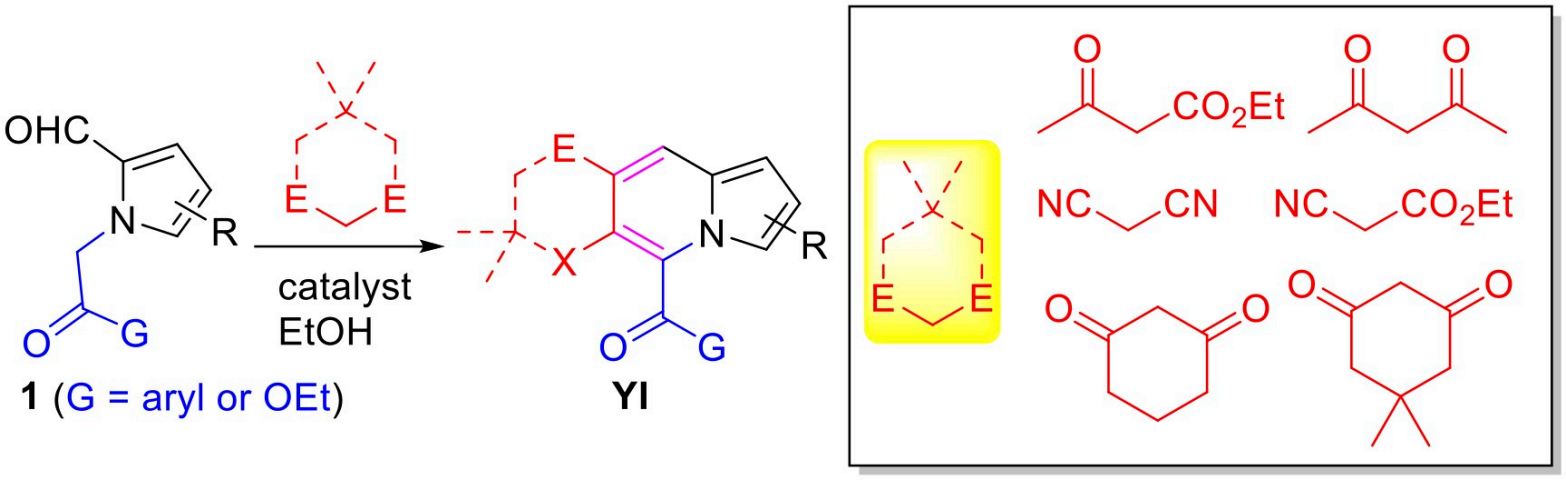
A scheme of synthesizing new indolizines.

Chemical reagents were purchased from Sigma-Aldrich (Missouri, United States). The indolizine-based chemical library was established using a domino Knoevenagel condensation-intramolecular aldol cyclization process, enabling access to novel indolizines with highly functionalized pyridines (Fig 6). [11] Thus, reaction in Fig 6 and several active methylene compounds in the presence of catalyst (piperidinium acetate, piperidine, or K_2_CO_3_) in EtOH at 120°C afforded a number of new indolizines (**YI**) in a diversity-oriented manner [11].

Compound **YI-10** was obtained in 88% yield as a result of incomplete dehydration when piperidine was used as a catalyst. Acid **YI-23** was prepared by hydrolysis of the corresponding ester **YI-24**. Aldehyde **YI-25** was produced via Vilsmeier-Haack formylation of **YI-24**. Enone **YI-26** was synthesized by Claisen-Schmidt aldol condensation of **YI-01** with 4-bromobenzaldehyde. Biological studies of this class of compounds were recorded previously [28, 29].

### Synthesis of YI-09

**Fig 7:**
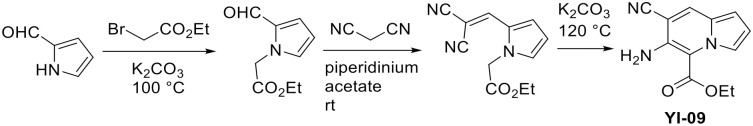
A scheme of synthesizing YI-09.

To a vial charged with pyrrole-2-carboxaldehyde (300 mg, 3.16 mmol) in acetonitrile (11 mL) were added ethyl bromoacetate (0.42 mL, 1.2 equiv) and potassium carbonate (567.33 mg, 1.3 equiv) at room temperature (rt) (Fig 7). After being stirred at 100°C for 3 hours, the reaction mixture was concentrated under reduced pressure, extracted with CH_2_Cl_2_ (5 mL), and washed with H_2_O (5 mL). The aqueous layer was extracted with CH_2_Cl_2_ (3 mL) two more times. The organic layer was dried over MgSO_4_, filtered, and concentrated *in vacuo*. The residue was purified by silica gel column chromatography (*n*-hexane:ethyl acetate:dichloromethane = 30:1:2) to give ethyl 2-(2-formyl-1*H*-pyrrol-1-yl)acetate (571.64 mg, 94%). To a solution of ethyl 2-(2-formyl-1*H*-pyrrol-1-yl)acetate (100 mg, 0.55 mmol) in ethanol (2 mL) were added malononitrile (54.7 mg, 1.5 equiv) and piperidinium acetate (39.9 mg, 0.5 equiv) at room temperature. After being stirred at room temperature for 16 hours, the reaction mixture was suction-filtered and dried to give ethyl 2-(2-(2,2-dicyanovinyl)-1*H*-pyrrol-1-yl)acetate (120 mg, 95%). To a solution of ethyl 2-(2-(2,2-dicyanovinyl)-1*H*-pyrrol-1-yl)acetate (100 mg, 0.44 mmol) in ethanol (6 mL) was added potassium carbonate (30.2 mg, 0.5 equiv) at room temperature. After being stirred at 120°C for 2 hours, the reaction mixture was concentrated *in vacuo*. The crude residue was diluted with CH_2_Cl_2_ (10 mL) and suction-filtered through a pad of Celite. The filtrate was purified by silica gel chromatography (*n*-hexane:ethyl acetate:dichloromethane = 10:1:2) give **YI-09** (51.8 mg, 52%).

#### Ethyl 6-amino-7-cyanoindolizine-5-carboxylate (YI-09)

Yellow solid, mp: 106.6–107.3°C; ^**1**^**H NMR** (400 MHz, CDCl_3_) δ 8.52 (s, 1H), 7.82 (s, 1H), 6.78 (d, *J* = 2.0 Hz, 2H), 6.39 (s, 2H), 4.51 (q, *J* = 7.2 Hz, 2H), 1.49 (t, *J* = 7.2 Hz, 3H); ^**13**^**C NMR** (100 MHz, CDCl_3_) δ 164.3, 142.7, 131.0, 128.5, 123.6, 116.8, 114.9, 109.1, 92.1, 61.4, 14.5; **HRMS** (ESI-QTOF) *m/z* [M+H]^+^ calcd for C_12_H_12_N_3_O_2_ 230.0924, found 230.0951.

### Synthesis of YI-10

**Fig 8:**
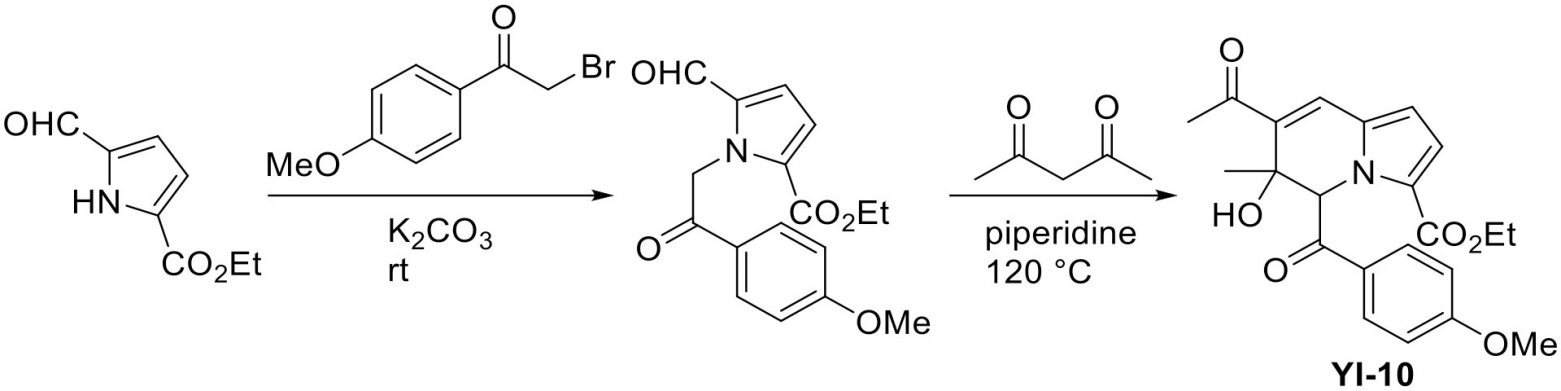
A scheme of synthesizing YI-10.

To a vial charged with ethyl 5-formyl-1*H*-pyrrole-2-carboxylate (100 mg, 0.598 mmol) in acetonitrile (2 mL) were added 2-bromo-4’-methoxyacetophenone (164.4 mg, 1.2 equiv) and potassium carbonate (124.02 mg, 1.5 equiv) at room temperature (rt) (Fig 8). After being stirred at room temperature for 16 hours, the reaction mixture was concentrated under reduced pressure, extracted with CH_2_Cl_2_ (5 mL), and washed with H_2_O (5 mL). The aqueous layer was extracted with CH_2_Cl_2_ (3 mL) two more times. The organic layer was dried over MgSO_4_, filtered, and concentrated *in vacuo*. The residue was purified by silica gel column chromatography (*n*-hexane:ethyl acetate:dichloromethane = 30:1:2) to give ethyl 5-formyl-1-(2-(4-methoxyphenyl)-2-oxoethyl)-1*H*-pyrrole-2-carboxylate (177.6 mg, 94%). To a solution of ethyl 5-formyl-1-(2-(4-methoxyphenyl)-2-oxoethyl)-1*H*-pyrrole-2-carboxylate (30 mg, 0.095 mmol) in ethanol (2 mL) were added acetylacetone (14.6 μL, 1.5 equiv) and piperidine (14.1 μL, 1.5 equiv) at room temperature. After being stirred at 120°C for 16 hours, the reaction mixture was concentrated *in vacuo*. The crude residue was purified by silica gel column chromatography (*n*-hexane:ethyl acetate:dichloromethane = 20:1:2) to give **YI-10** (31.6 mg, 88%).

#### Ethyl 7-acetyl-6-hydroxy-5-(4-methoxybenzoyl)-6-methyl-5,6-dihydroindolizine-3-carboxylate (YI-10)

Pale yellow solid, mp: 178.7–179.1°C; ^**1**^**H NMR** (400 MHz, CDCl_3_) δ 8.10 (d, *J* = 8.8 Hz, 2H), 7.40 (s, 1H), 7.03 (d, *J* = 9.2 Hz, 1H), 6.96 (d, *J* = 9.2 Hz, 2H), 6.86 (s, 1H), 6.57 (d, *J* = 4.0 Hz, 1H), 5.86 (s, 1H), 4.08–4.27 (m, 2H), 3.87 (s, 3H), 2.41 (s, 3H), 1.62 (s, 3H), 1.24 (t, *J* = 7.2 Hz, 3H); ^**13**^**C NMR** (100 MHz, CDCl_3_) δ 200.8, 193.3, 163.5, 160.5, 133.6, 132.1, 131.5, 130.5, 128.8, 126.0, 119.5, 113.9, 113.5, 74.8, 63.7, 60.5, 55.4, 30.2, 25.9, 14.2; **HRMS** (ESI-QTOF) *m/z* [M+H]^+^ calcd for C_22_H_24_NO_6_ 398. 1598, found 398.1671.

### Synthesis of YI-23

**Fig 9:**
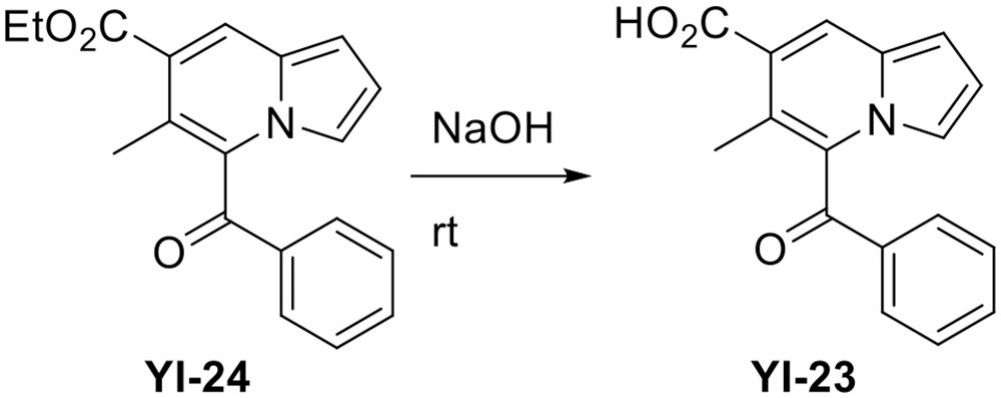
A scheme of synthesizing YI-23.

To a solution of **YI-24** (124.7 mg, 0.41 mmol) in MeOH/H_2_O (1:1, 1.5 mL) was added NaOH (162.28 mg, 10.0 equiv) at room temperature (rt) (Fig 9). After being stirred at room temperature for 16 hours, the reaction mixture was concentrated *in vacuo* and neutralized with 10% HCl. The resulting precipitate was suction-filtered and dried to give **YI-23** (114.5 mg, 100%).

#### 5-Benzoyl-6-methylindolizine-7-carboxylic acid (YI-23)

Greenish yellow solid, mp: 179.8–180.4°C; ^**1**^**H NMR** (400 MHz, CDCl_3_) δ 8.49 (s, 1H), 7.91 (d, *J* = 8.8 Hz, 2H), 7.67 (t, *J* = 7.2 Hz, 1H), 7.50 (t, *J* = 7.6 Hz, 2H), 7.07 (s, 1H), 6.79–6.85 (m, 2H), 2.37 (s, 3H); ^**13**^**C NMR** (100 MHz, CDCl_3_) δ 192.9, 172.1, 135.4, 135.0, 130.9, 130.3, 129.6, 129.4, 126.6, 117.8, 117.5, 115.9, 115.1, 105.9, 16.9; **HRMS** (ESI-QTOF) *m/z* [M+H]^+^ calcd for C_17_H_14_NO_3_ 280.0968, found 280.1007.

### ThT fluorescence assay

ThT fluorescence assay was conducted to confirm Aβ fibrilization and to quantify the β-sheet complex of Aβ aggregates [17]. Previously reported DMSO-incorporated Fmoc solid phase peptide synthesis protocol was applied to synthesize Aβ42 peptides [30]. In-house synthetic Aβ42 peptides were dissolved in DMSO (1 mM), purchased from Sigma-Aldrich (Missouri, USA), and distilled with deionized water to make Aβ stock solution (100 μM). During the inhibition assay, indolizine derivatives dissolved in DMSO and diluted with deionized water to three different concentrations (0.5, 5, and 50 μM) were incubated with monomeric Aβ42 (final concentration of 50 μM) at 37°C for three days. During the disaggregation assay, Aβ42 stock only was incubated at 37°C for three days to initially prepare Aβ aggregates. Then, Aβ aggregates were mixed with indolizine derivatives (same concentrations as above) and reincubated for additional three days at 37°C. After the incubation, 25 μL of the samples and 75 μL of ThT solution (5 μM ThT in 50 mM glycine buffer, pH 8.9) were loaded in a 96-well half area black plate. ThT was purchased from Sigma-Aldrich (Missouri, USA) and 96-well half area black plate was purchased from Corning (New York, USA). Fluorescent intensities of ThT bound to Aβ were measured at 450 nm (excitation) and 485 nm (emission) by using a microplate reader (Infinite 200 PRO, Tecan).

### SDS-PAGE with PICUP

PICUP and SDS-PAGE analysis were performed to evaluate the amounts of Aβ oligomers, protofibrils, and fibrils [31–33]. For inhibition analysis, indolizine derivatives dissolved in DMSO and diluted with distilled water (250 μM) were incubated with monomeric Aβ42 (final concentration of 50 μM) at 37°C for three days. For disaggregation analysis, Aβ peptide (100 μM) incubated by itself for three days at 37°C was reincubated after the addition of indolizine derivatives (250 μM) for additional three days at the same temperature. For Aβ peptides cross-linking, 10 mM Tris(2,2′-bipyridyl)dichlororuthenium(II) hexahydrate (Ru(Bpy)) and 200 mM Ammonium persulfate (APS) were dissolved in buffer A (0.1 M sodium phosphate, pH 7.4), and they were diluted with the same buffer to make 1 mM and 20 mM respectively. Both Ru(Bpy) and APS were purchased from Sigma-Aldrich, USA. Then, 1 μL of both 1 mM Ru(Bpy) and 20 mM APS were added to 10 μL of each incubated sample. The mixed solutions were irradiated by visible light for three seconds with 1 second break between each second, and the reaction was quenched by adding 3 μL of 5X sample buffer with β-mercaptoethanol (Sigma-Aldrich, USA). The samples were further boiled for five minutes at 95°C, and peptides were separated by SDS-PAGE electrophoresis on 1.0 mm-thick 15% gradient polyacrylamide gel (Gradi-Gel II, ELPIS). After the gel running, Aβ bands separated by different sizes were visualized by silver staining according to the PlusOne Silver Staining Kit protocol (GE Healthcare, USA).

### Fluorescent spectral scan

Every fluorescent spectral scan performed in this study was done under the microplate reader (Infinite 200 PRO, Tecan). To acquire the excitation point of each indolizine-derived compounds, the derivatives were dissolved in DMSO and diluted with deionized water in final concentration of 250 μM. Then, 150 μL of each compound solution was loaded into a 96-well clear round-bottom plate (Corning, USA) and measured at 2 nm increments from 230 nm to 850 nm. The peaks for each compound through absorbance scan were indicated as possible excitation wavelengths. To obtain an emission wavelength, 150 μL of each compound solution, prepared same as above, was transferred to a 96-well opaque round-bottom plate (Greiner Bio-One, Austria). By applying the respective excitation wavelength for each compound, the emission scans were recorded at 2 nm increments between the ranges from 20 nm added to the absorption scan maximum to 850 nm. The narrowest and highest peak exhibited through emission scans of each compound was determined as its emission point. Using the final excitation and emission points obtained from the absorbance and emission scans, the fluorescent intensities of candidate compounds when incubated with Aβ were scanned to observe whether these compounds show a fluorescence shift in the presence of Aβ. Aβ monomers were prepared by storing Aβ42 peptides, dissolved in DMSO (1 mM) followed by serial dilution with distilled water (25 μM), at –80°C. On the other hand, Aβ aggregates were prepared by incubating the same Aβ as above at 37°C for three days. Indolizine derivatives were also dissolved in DMSO and diluted with distilled water in final concentration of 250 μM. Then, 25 μL of either Aβ monomers or aggregates (25 μM) was mixed with 75 μL of indolizine derivatives (250 μM) in each well of a 96-well opaque round-bottom plate. The emission spectra of each compound in the presence of Aβ were recorded at 2 nm increments at varying spectra ranges.

### Animals

Transgenic mouse (strain name; B6SJL-Tg(APPSwF1Lon, PSEN1*M146L*L286V) 6799Vas/Mmjax) carrying five mutations associated with early onset familial Alzheimer’s disease (5XFAD) was used throughout this experiment. The 5XFAD mice were acquired from Jackson Laboratory (Maine, USA) and have been conserved through mating with C57BL/6 X SJL wild type mice. All mice were bred in a laboratory animal breeding room at Yonsei University (Seoul, South Korea) under regulated conditions with 12hour/12hour light-dark phase. Food and Water were provided *ad libitum*. All animal experiments were conducted in accordance with the National Institutes of Health (NIH) Guide for the Care and Use of Laboratory Animals. The research protocols were authorized by the Institutional Animal Care and Use Committee of Yonsei University.

### Histochemistry of 5XFAD mouse brain

Brain Tissues were fixed in 4% paraformaldehyde (Biosesang, South Korea) overnight at 4°C and immersed in 30% sucrose for 48 hours for cryoprotection. Brain sections (35 μm) were cut with a cryostat (CM1860, Leica) and attached to slides. The antigen retrieval on fixed brain sections was conducted using 1% SDS (Biosesang, South Korea) in 1X PBS (Gibco, South Korea) for 10 minutes, followed by blocking with 20% horse serum in 1X PBS for an hour. Then, we incubated the slides at 4°C overnight with mouse monoclonal antibody 6E10 (1:200, BioLegend, USA), known as the primary antibody that detects Aβ plaques. On the next day, the slides were incubated with goat anti-mouse IgG conjugated with Alexa Fluor Plus 488 (1:200, Life Technologies, USA) for an hour at room temperature. Indolizine derivatives stock in DMSO (10 mM) were diluted 500 μM with 1X PBS and added to the brain tissues for seven minutes at room temperature followed by rinsing with 90, 70, and 50% ethanol and PBS in order. The amyloid plaques in the cortex and the hippocampus of the fixed brain sections were visualized under a fluorescence microscope (DM500, Leica), provided with filter cubes containing excitation and emission filters: N2.1 filter cube for 6E10 staining detection (excitation filter: BP 515–560; dichromatic mirror: 580; emission filter: LP 590) and L5 filter cube for YQ compound detection (excitation filter: BP 480/40; dichromatic mirror: 505; emission filter: BP 527/30).

### Lysate analysis

To prepare brain lysates, mice were sacrificed, and hippocampal and cortical regions of mouse brains were dissected separately. Each brain region was homogenized in ice-cold RIPA buffer (20 mM Tris-HCl, pH 7.5, 50 mM NaCl, 0.5% NP-40, 4 mM EDTA, 0.1% SDS, 0.5% sodium deoxycholate) containing 1X protease inhibitor cocktail (Roche Diagnostics, Switzerland) [34]. Homogenized brain tissues were incubated in ice for 20 minutes before centrifugation at 14,000 rpm at 4°C for 30 minutes. The supernatants (soluble fraction) of brain lysates were collected. To obtain Aβ-insoluble fraction in brain lysates, the pellet was rehomogenized with a guanidine buffer (5 mM GdnHCl, 50 mM Tris-HCl, pH 8.0) containing 1X proteinase inhibitor cocktail. The mixtures were incubated at room temperature for 3 hours on multi mixer to dissolve Aβ-insoluble fraction, and they were centrifuged at 14,000 rpm at 4°C for 2 hours. The supernatant (insoluble fraction) of brain lysates was collected.

In order to analyze **YI-13**’s interaction with mice brain lysate samples, we treated 10 μL of brain lysates, either soluble or insoluble, from hippocampal and cortical regions with 10 μL of 10X protease inhibitors and diluted the mixture with 80 μL of 1X PBS. The **YI-13** solution was diluted to 250 μM with 1X PBS. The individual brain lysate sample and **YI-13** were loaded to the wells of a 96-well half-area black microplate (Corning, USA) with a sample to compound ratio of 1:3. The samples were distributed in triplicates and detected at λ_ex_ = 394 nm/λ_em_ = 582 nm.

### Dot blot assay

The dot blot assay was performed to confirm Aβ42 oligomer formation in both the cortical and hippocampal regions of mice brains [35]. The same brain lysate samples mentioned above were used in this assay. The protein concentrations in supernatants were quantified via Pierce^™^ BCA protein assay kit (Thermo Fisher Scientific, USA). Briefly, 20 μg of brain lysates were loaded on a nitrocellulose membrane and dried for 30 minutes. Then, 6E10 (1:1,000, BioLegend, USA) and antibody were used to detect Aβ oligomers. After the overnight incubation, membranes were incubated with HRP-conjugated goat anti-mouse secondary antibody (1:10,000, Bethyl Laboratories, USA). All washes were performed with TBS-T, three times for five minutes, except for the last wash which took for 10 minutes.

### MWCO filtration of Aβ oligomers

MWCO filters were utilized to isolate Aβ oligomers from heterogeneous mixture of Aβ aggregates. Amicon^®^ Ultra centrifugation filters (Merck Millipore, USA) of 100K cut-off was used to separate bigger size of Aβ aggregates, considered to be fibrils. Then, Vivaspin 500 centrifugal concentrators (Sigma-Aldrich, USA) of 30K cut-off was used to separate oligomers (about 15 to 75 kDa) from smaller monomers (5 kDa). To examine **YI-13**’s interaction with the oligomers, we added 25 μL of filtered Aβ oligomers (25 μM) and 75 μL of **YI-13** (250 μM) in each well of a 96-well opaque round-bottom plate (Corning, USA). The **YI-13** solution was diluted the same way as it was in fluorescent spectral scan. The emission spectra of **YI-13** was recorded at 2 nm increments with λ_ex_ = 394 nm/λ_em_ = 582 nm.

### Syntheses of Aβ42 and Aβ dimers

Full-length Aβ42 and Aβ dimers were synthesized by modified Fmoc solid-phase peptide synthesis protocols as previously reported [25, 30]. In order to observe **YI-13**’s alterations in fluorescent intensity when added to Aβ dimers, Aβ was first dissolved in DMSO (1 mM) followed by serial dilution with distilled water (25 μM). Then, 25 μL of Aβ dimers (25 μM) was mixed with 75 μL of **YI-13** (250 μM) in each well of a 96-well opaque round-bottom plate (Corning, USA). The emission spectra of **YI-13** was recorded at 2 nm increments with λ_ex_ = 394 nm/λ_em_ = 582 nm.

### Measurements of YI-13 fluorescent properties

#### Excitation and emission maximum measurements

Every measurement performed in this study was done under Cary3500 compact UV-Vis (Agilen, USA) for absorbance scan and LS-55 Fluorescence Spectrometer (PerkinElmer, USA) for fluorescent intensity scan. **YI-13** was initially dissolved in DMSO and diluted with deionized water in final concentration of 250 μM. For **YI-13** only measurement, 3 mL of **YI-13** (250 μM) was read for its absorbance and emission to analyze excitation/emission maximum. To evaluate the excitation and emission maximum of **YI-13** in the presence of Aβ, **YI-13** and Aβ monomers (0d)/aggregates (3d), prepared the same way as above, were added in the ratio of 3 to 1 in total volume of 3 mL.

#### Extinction, quantum yield, and brightness analysis

Every measurement performed in this study was done under Cary3500 compact UV-Vis (Agilen, USA) for absorbance scan and LS-55 Fluorescence Spectrometer (PerkinElmer, USA) for fluorescent intensity scan. **YI-13** solution was prepared by dissolving in DMSO and diluting with deionized water in final concentration of 500 μM. For **YI-13** added with Aβ solutions, **YI-13** (500 μM) was added to Aβ monomers or aggregates (both 25 μM) in the ratio of 3 to 1 in total volume of 3 mL. Then, absorbance and fluorescent intensities were measured in succession by serial dilution of mixture solution by half. Extinction coefficient values were calculated according to the Beer-Lambert law [36]. Quantum yield was evaluated by comparing slope of absorbance to fluorescence with slope of Rhodamine 6, set as a control [37]. Brightness was quantified by multiplying quantum yield to molar absorption coefficient.

### Statistical analysis

All graphs were obtained with GraphPad Prism 7.0 software, and all statistical analyses were conducted with one-way ANOVA followed by Bonferroni’s posthoc comparisons (*P < 0.033, **P < 0.002, ***P < 0.001). The error bars represent the standard error of the mean (SEM).

## Supporting information

S1 Fig Denormalized data of ThT assay to confirm anti-Aβ aggregation activity of indolizine-derived YI compounds. ThT fluorescence assay was conducted for (A) inhibition of Aβ aggregation and (B) disaggregation of pre-formed Aβ aggregation by using 50 μM Aβ42 with 0.5, 5, and 50 μM **YI** compounds as shown. The samples of Aβ42 added to the compound were incubated for three days (3d) in total for inhibition tests and six days (6d) in total for disaggregation tests. Abbreviations: 0d = Aβ monomers, 3d = 3-day incubation of Aβ, 6d = 3-day pre-incubation of Aβ and additional 3-day incubation of Aβ. Data represents the mean of triplicated experiments ± SEMs and one-way anova was applied followed by Bonferoni’s post-hoc comparison test (*P < 0.033, **P < 0.002, ***P < 0.001).
(DOCX)

S2 Fig Full image of SDS-PAGE analysis to confirm anti-amyloidogenic properties of indolizine-derived YI compounds, related to Fig 2C. Full-length original gels of SDS-PAGE with PICUP and silver staining for disaggregation of Aβ42 (50 μM, 3-day pre-aggregation) aggregates by **YI** compounds (250 μM). Sizes of Aβ species according to size markers are monomers (5 kDa), dimers (10 kDa), oligomers (15 to 75 kDa), and larger aggregates or fibrils (embedded at the top of the gels). Abbreviations: + = 3-day incubation of Aβ, ++ = 3-day pre-incubation of Aβ and additional 3-day incubation of Aβ and/or compounds.
(DOCX)

S3 Fig Fluorescence spectroscopy of selected 15 indolizine-derived YI compounds without presence of Aβ aggregates. We recorded (A) absorbance spectra and (B) emission spectra of the selected 15 of the novel indolizine derivatives to obtain the excitation and emission wavelength. The highest peak of the spectrum in (A) indicates the excitation wavelength of each compound, and they are as following: **YI-01**, 362 nm; **YI-02**, 472 nm; **YI-03**, 330 nm; **YI-04**, 412 nm; **YI-05**, 320 nm; **YI-07**, 332 nm; **YI-08**, 310 nm; **YI-12**, 410 nm; **YI-13**, 394 nm; **YI-14**, 440 nm; **YI-15**, 475 nm; **YI-16**, 330 nm; **YI-17**, 486 nm; **YI-22**, 480 nm; **YI-26**, 415 nm. The highest peak of the spectrum in (B) indicates the emission wavelength of each compound, and they are as following: **YI-01**, 500 nm; **YI-02**, 610 nm; **YI-03**, 585 nm; **YI-04**, 620 nm; **YI-05**, 445 and 500 nm; **YI-07**, 427 nm; **YI-08**, 622 nm; **YI-12**, 486 nm; **YI-13**, 582 nm; **YI-14**, 604 nm; **YI-15**, 529 nm; **YI-16**, 555 nm; **YI-17**, 604 nm; **YI-22**, 610 nm; **YI-26**, 579 nm. These excitation and emission wavelengths were applied when measuring the fluorescence spectral scan of 15 compounds in the presence of Aβ aggregates. Abbreviations: FI = fluorescence intensity, A.U. = arbitrary unit.
(DOCX)

S4 Fig Histochemical analyses of 6E10 and YI-13 on two separate brain tissue sections obtained from aged male 5XFAD transgenic mouse model. Two consecutive brain tissue sections were acquired through cryostat and each section was stained with 6E10 and **YI-13** respectively due to the overlapping wavelength range of 6E10 and **YI-13**. The arrows demonstrate that both 6E10 and **YI-13** co-localize Aβ plaques in 5XFAD mouse model. Scale bars = 500 μm. Abbreviations: HIP = hippocampus, CTX = cortex.
(DOCX)

S5 Fig Full image of dot blot assay to compare total Aβ levels in the hippocampus and cortex of the 5XFAD transgenic mouse model, related Fig 4D. Soluble Aβ oligomers were applied to a nitrocellulose membrane and probed with 6E10 which recognizes all species of Aβ. Abbreviations: WT = wild-type, TG = transgenic.
(DOCX)

S6 Fig Histochemical analyses of WT littermates with 6E10 and YI-13, related to Fig 4B. Aβ deposition stained with 6E10 and YI-13 in either hippocampal (up) or cortical (down) region. The merged images of 6E10 and YI-13 staining are also shown. Scale bars, 100 μm.
(DOCX)

S1 Raw images (PDF)
