# Correction: Fluorescent indolizine derivative YI-13 detects amyloid-β monomers, dimers, and plaques in the brain of 5XFAD Alzheimer transgenic mouse model

**DOI:** 10.1371/journal.pone.0250194

**Published:** 2021-04-08

**Authors:** DaWon Kim, Jeong Hwa Lee, Hye Yun Kim, Jisu Shin, Kyeonghwan Kim, Sejin Lee, Jinwoo Park, Ikyon Kim, YoungSoo Kim

The eighth author’s name is spelled incorrectly in the byline and in the Resources subsection of the Author Contributions. The correct name is: Ikyon Kim.

The eighth author’s initials are listed incorrectly after their email address. The correct initials are: IK.
