# Supplementary material for: Fluorescent indolizine derivative YI-13 detects amyloid-β monomers, dimers, and plaques in the brain of 5XFAD Alzheimer transgenic mouse model
Source: PLoS One. 2020 Dec 23;15(12):e0243041. doi: 10.1371/journal.pone.0243041 (PMC7757811; doi:10.1371/journal.pone.0243041)
Supplement: S1 Fig — ThT fluorescence assay was conducted for (A) inhibition of Aβ aggregation and (B) disaggregation of pre-formed Aβ aggregation by using 50 μM Aβ42 with 0.5, 5, and 50 μM YI compounds as shown. The samples of Aβ42 added to the compound were incubated for three days (3d) in total for inhibition tests and six days (6d) in total for disaggregation tests. Abbreviations: 0d = Aβ monomers, 3d = 3-day incubation of Aβ, 6d = 3-day pre-incubation of Aβ and additional 3-day incubation of Aβ. Data represents the mean of triplicated experiments ± SEMs and one-way anova was applied followed by Bonferoni’s post-hoc comparison test (*P < 0.033, **P < 0.002, ***P < 0.001). (DOCX) [file pone.0243041.s001.docx]

**
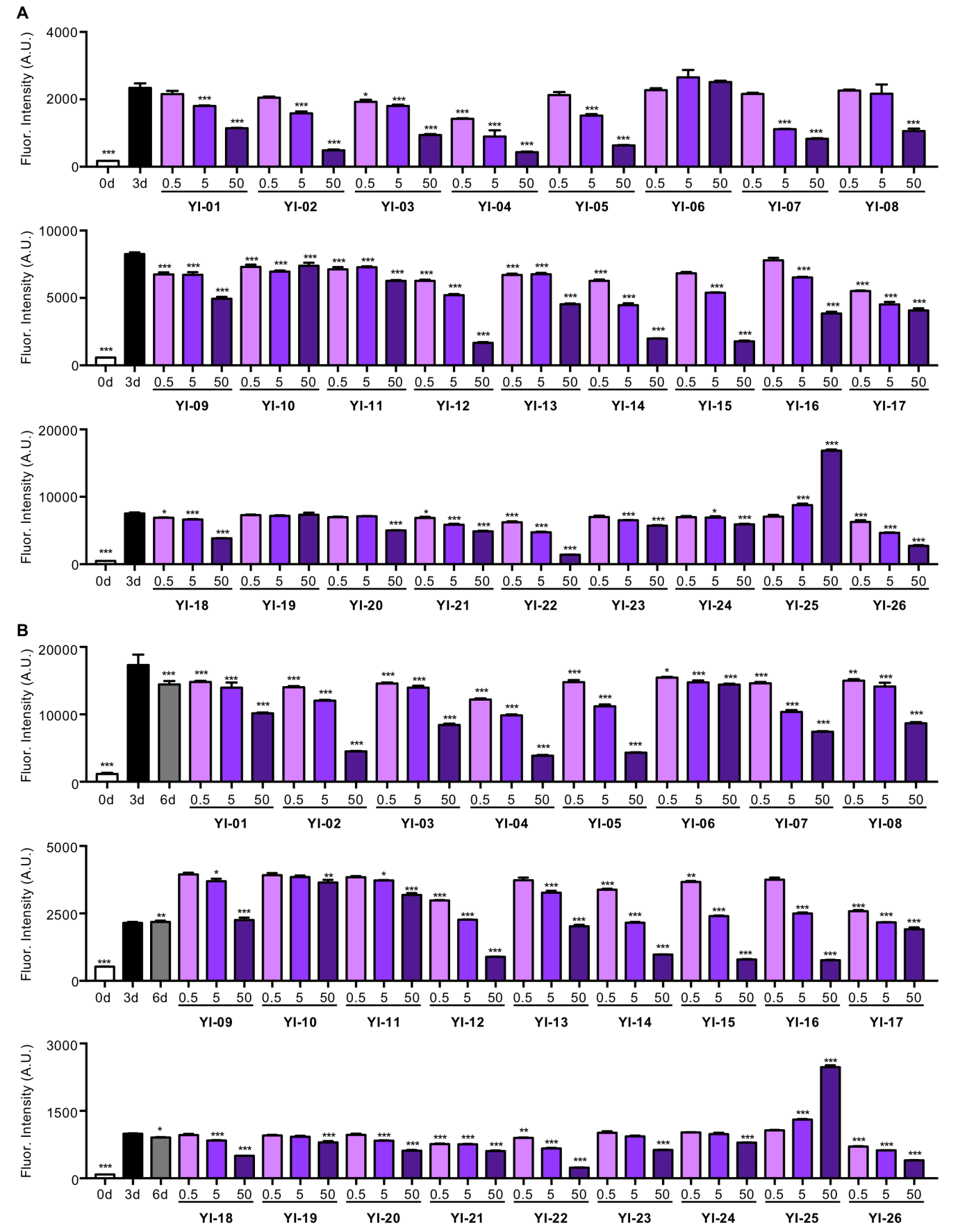
**

**S1 Fig. Denormalized data of ThT assay to confirm anti-Aβ aggregation activity of indolizine-derived YI compounds.** ThT fluorescence assay was conducted for (A) inhibition of Aβ aggregation and (B) disaggregation of pre-formed Aβ aggregation by using 50 μM Aβ42 with 0.5, 5, and 50 μM YI compounds as shown. The samples of Aβ42 added to the compound were incubated for three days (3d) in total for inhibition tests and six days (6d) in total for disaggregation tests. Abbreviations: 0d = Aβ monomers, 3d = 3-day incubation of Aβ, 6d = 3-day pre-incubation of Aβ and additional 3-day incubation of Aβ, A.U. = arbitrary unit. Data represents the mean of triplicated experiments ± SEMs and one-way anova was applied followed by Bonferoni’s post-hoc comparison test (*P < 0.033, **P < 0.002, ***P < 0.001).
