# Supplementary material for: Fluorescent indolizine derivative YI-13 detects amyloid-β monomers, dimers, and plaques in the brain of 5XFAD Alzheimer transgenic mouse model
Source: PLoS One. 2020 Dec 23;15(12):e0243041. doi: 10.1371/journal.pone.0243041 (PMC7757811; doi:10.1371/journal.pone.0243041)
Supplement: S3 Fig — We recorded (A) absorbance spectra and (B) emission spectra of the selected 15 of the novel indolizine derivatives to obtain the excitation and emission wavelength. The highest peak of the spectrum in (A) indicates the excitation wavelength of each compound, and they are as following: YI-01, 362 nm; YI-02, 472 nm; YI-03, 330 nm; YI-04, 412 nm; YI-05, 320 nm; YI-07, 332 nm; YI-08, 310 nm; YI-12, 410 nm; YI-13, 394 nm; YI-14, 440 nm; YI-15, 475 nm; YI-16, 330 nm; YI-17, 486 nm; YI-22, 480 nm; YI-26, 415 nm. The highest peak of the spectrum in (B) indicates the emission wavelength of each compound, and they are as following: YI-01, 500 nm; YI-02, 610 nm; YI-03, 585 nm; YI-04, 620 nm; YI-05, 445 and 500 nm; YI-07, 427 nm; YI-08, 622 nm; YI-12, 486 nm; YI-13, 582 nm; YI-14, 604 nm; YI-15, 529 nm; YI-16, 555 nm; YI-17, 604 nm; YI-22, 610 nm; YI-26, 579 nm. These excitation and emission wavelengths were applied when measuring the fluorescence spectral scan of 15 compounds in the presence of Aβ aggregates. Abbreviations: FI = fluorescence intensity, A.U. = arbitrary unit. (DOCX) [file pone.0243041.s003.docx]

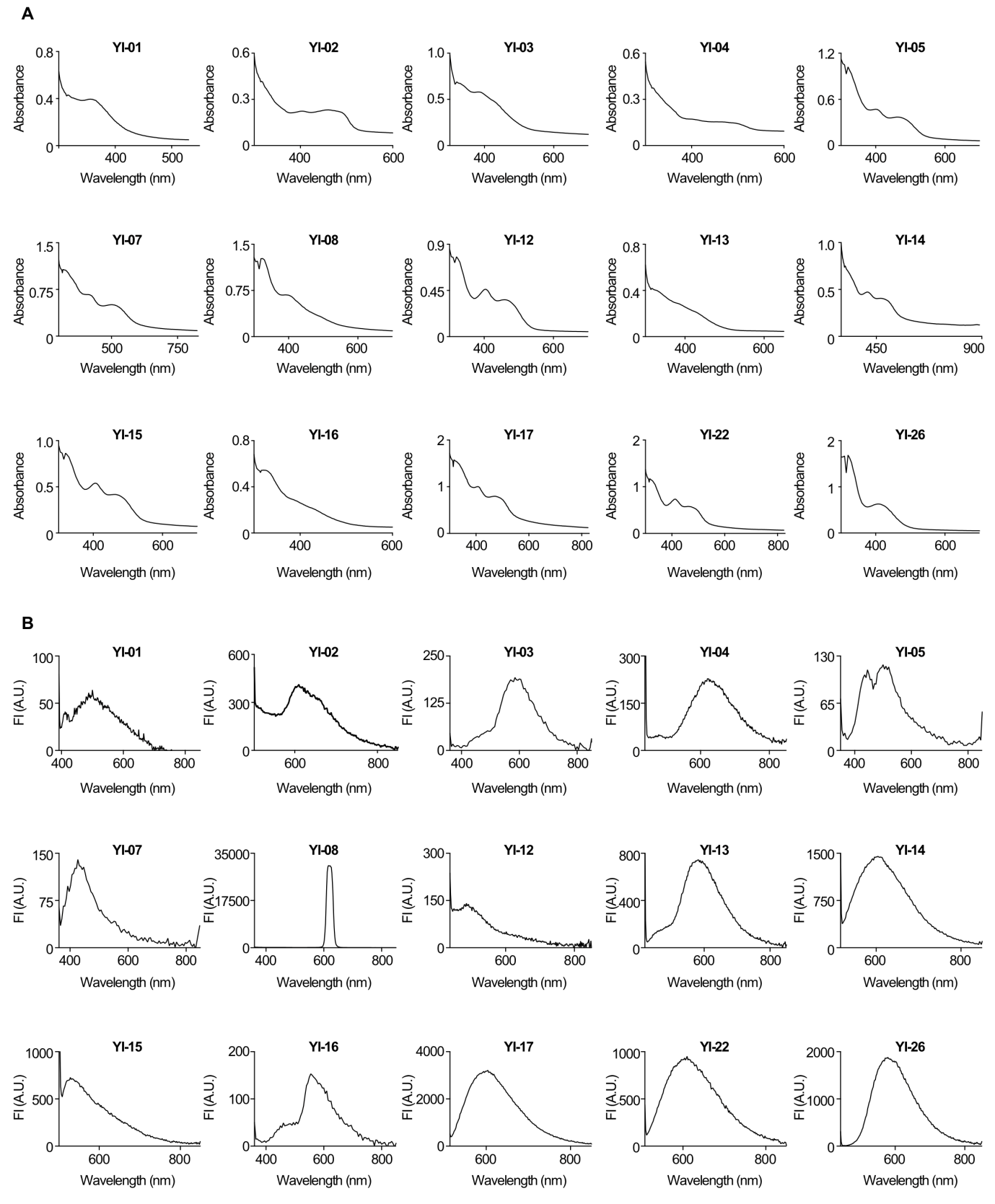


**S3 Fig. Fluorescence spectroscopy of selected 15 indolizine-derived YI compounds without presence of Aβ aggregates.** We recorded (A) absorbance spectra and (B) emission spectra of the selected 15 of the novel indolizine derivatives to obtain the excitation and emission wavelength. The highest peak of the spectrum in (A) indicates the excitation wavelength of each compound, and they are as following: YI-01, 362 nm; YI-02, 472 nm; YI-03, 330 nm; YI-04, 412 nm; YI-05, 320 nm; YI-07, 332 nm; YI-08, 310 nm; YI-12, 410 nm; YI-13, 394 nm; YI-14, 440 nm; YI-15, 475 nm; YI-16, 330 nm; YI-17, 486 nm; YI-22, 480 nm; YI-26, 415 nm. The highest peak of the spectrum in (B) indicates the emission wavelength of each compound, and they are as following: YI-01, 500 nm; YI-02, 610 nm; YI-03, 585 nm; YI-04, 620 nm; YI-05, 445 and 500 nm; YI-07, 427 nm; YI-08, 622 nm; YI-12, 486 nm; YI-13, 582 nm; YI-14, 604 nm; YI-15, 529 nm; YI-16, 555 nm; YI-17, 604 nm; YI-22, 610 nm; YI-26, 579 nm. These excitation and emission wavelengths were applied when measuring the fluorescence spectral scan of 15 compounds in the presence of Aβ aggregates. Spectra of all samples were acquired using an Infinite 200 PRO plate reader. Abbreviation: FI = fluorescence intensity, A.U. = arbitrary unit.
