# Supplementary material for: Fluorescent indolizine derivative YI-13 detects amyloid-β monomers, dimers, and plaques in the brain of 5XFAD Alzheimer transgenic mouse model
Source: PLoS One. 2020 Dec 23;15(12):e0243041. doi: 10.1371/journal.pone.0243041 (PMC7757811; doi:10.1371/journal.pone.0243041)
Supplement: S4 Fig — Two consecutive brain tissue sections were acquired through cryostat and each section was stained with 6E10 and YI-13 respectively due to the overlapping wavelength range of 6E10 and YI-13. The arrows demonstrate that both 6E10 and YI-13 co-localize Aβ plaques in 5XFAD mouse model. Scale bars = 500 μm. Abbreviations: HIP = hippocampus, CTX = cortex. (DOCX) [file pone.0243041.s004.docx]

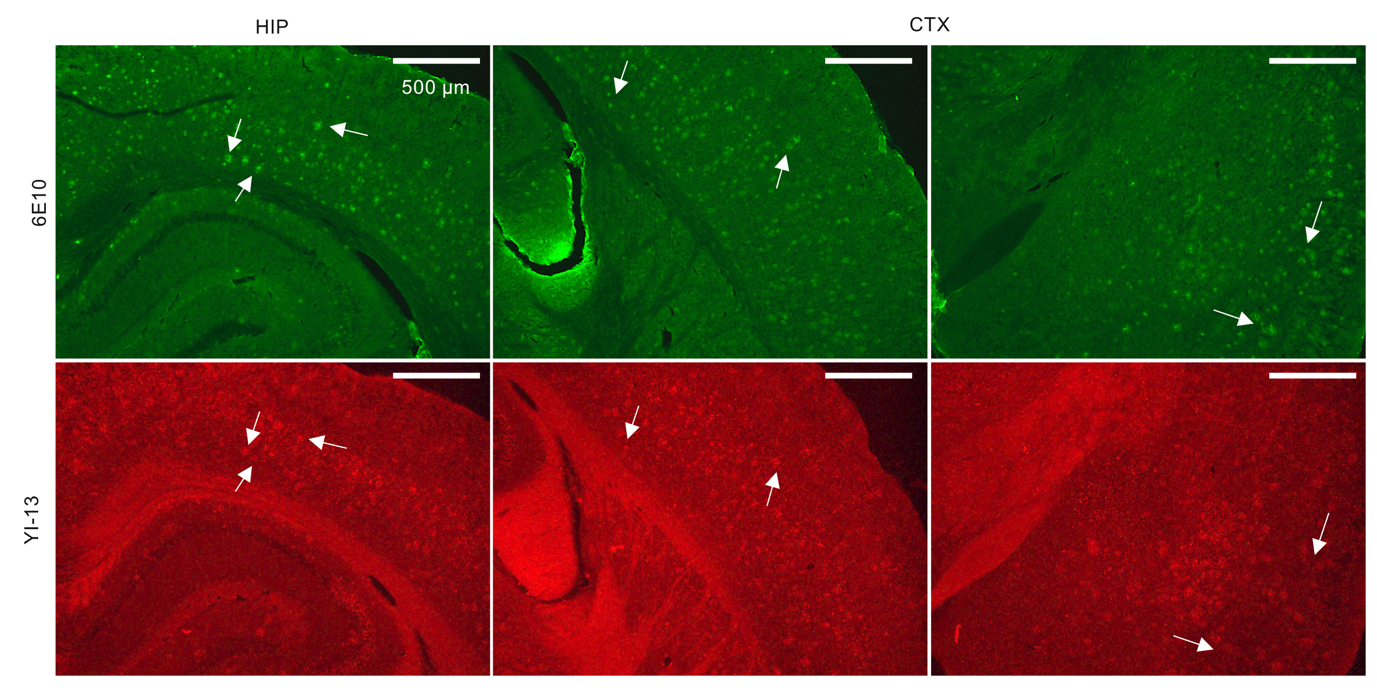


**S4 Fig. Histochemical analyses of 6E10 and YI-13 on two separate brain tissue sections obtained from aged male 5XFAD transgenic mouse model.** Two consecutive brain tissue sections were acquired through cryostat and each section was stained with 6E10 and YI-13 respectively due to the overlapping wavelength range of 6E10 and YI-13. The arrows demonstrate that both 6E10 and YI-13 co-localize Aβ plaques in 5XFAD mouse model. Scale bars = 500 μm. Abbreviations: HIP = hippocampus, CTX = cortex.
