# Supplementary material for: Fluorescent indolizine derivative YI-13 detects amyloid-β monomers, dimers, and plaques in the brain of 5XFAD Alzheimer transgenic mouse model
Source: PLoS One. 2020 Dec 23;15(12):e0243041. doi: 10.1371/journal.pone.0243041 (PMC7757811; doi:10.1371/journal.pone.0243041)
Supplement: S5 Fig — Soluble Aβ oligomers were applied to a nitrocellulose membrane and probed with 6E10 which recognizes all species of Aβ. Abbreviations: WT = wild-type, TG = transgenic. (DOCX) [file pone.0243041.s005.docx]

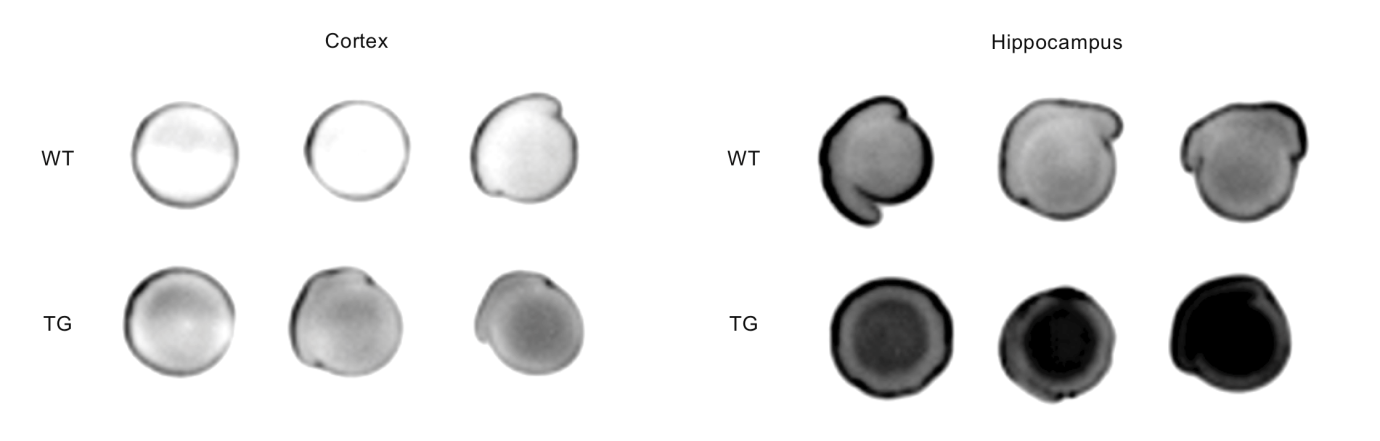


**S5 Fig. Full image of dot blot assay to compare total Aβ levels in the hippocampus and cortex of the 5XFAD transgenic mouse model.** Soluble Aβ oligomers were applied to a nitrocellulose membrane and probed with 6E10 which recognizes all species of Aβ. Abbreviations: WT = wild-type, TG = transgenic.
