# Supplementary material for: Fluorescent indolizine derivative YI-13 detects amyloid-β monomers, dimers, and plaques in the brain of 5XFAD Alzheimer transgenic mouse model
Source: PLoS One. 2020 Dec 23;15(12):e0243041. doi: 10.1371/journal.pone.0243041 (PMC7757811; doi:10.1371/journal.pone.0243041)
Supplement: S6 Fig — Aβ deposition stained with 6E10 and YI-13 in either hippocampal (up) or cortical (down) region. The merged images of 6E10 and YI-13 staining are also shown. Scale bars, 100 μm. (DOCX) [file pone.0243041.s006.docx]

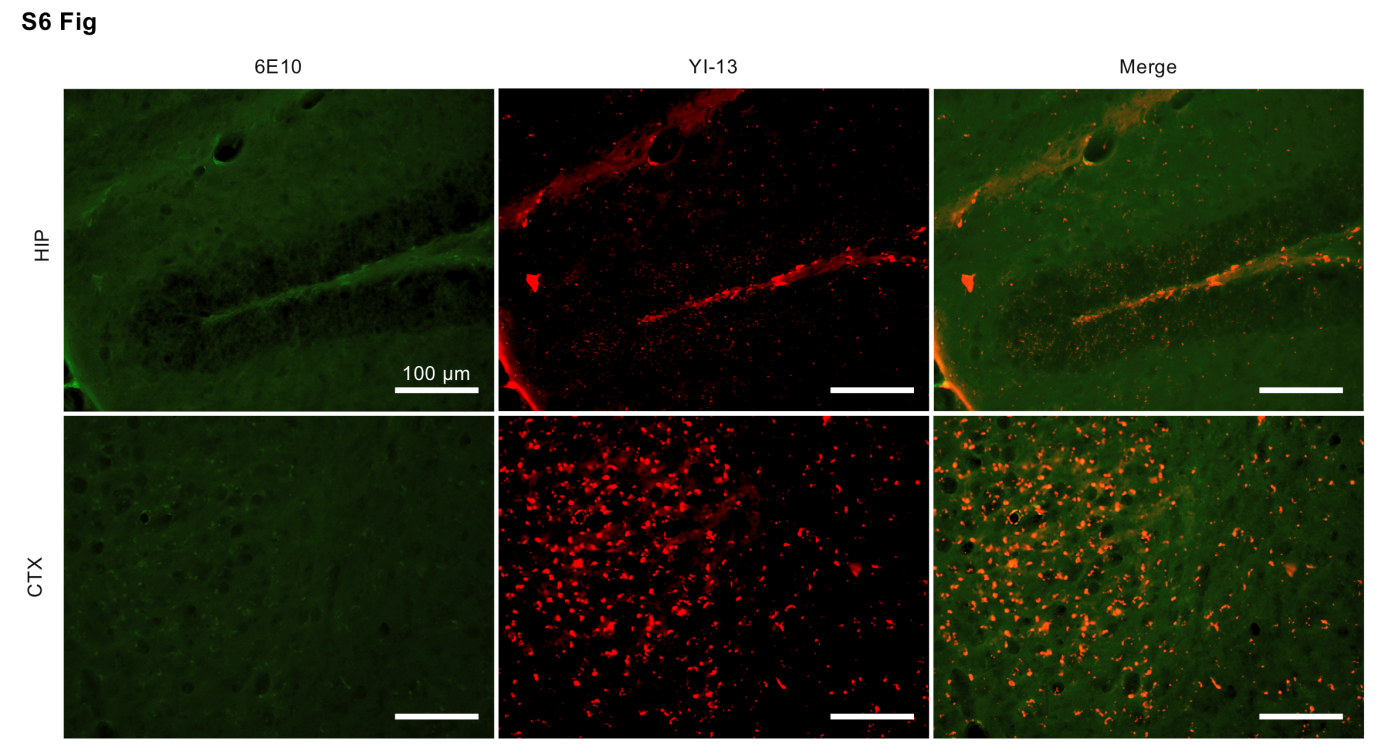


**S6 Fig. Histochemical analyses of WT littermates with 6E10 and YI-13, related to Fig 4B.** Aβ deposition stained with 6E10 and YI-13 in either hippocampal (up) or cortical (down) region. The merged images of 6E10 and YI-13 staining are also shown. Scale bars, 100 μm.
