# Supplementary material for: Fluorescent indolizine derivative YI-13 detects amyloid-β monomers, dimers, and plaques in the brain of 5XFAD Alzheimer transgenic mouse model
Source: PLoS One. 2020 Dec 23;15(12):e0243041. doi: 10.1371/journal.pone.0243041 (PMC7757811; doi:10.1371/journal.pone.0243041)
Supplement: S1 Raw images — (PDF) [file pone.0243041.s007.pdf]

Raw image 1

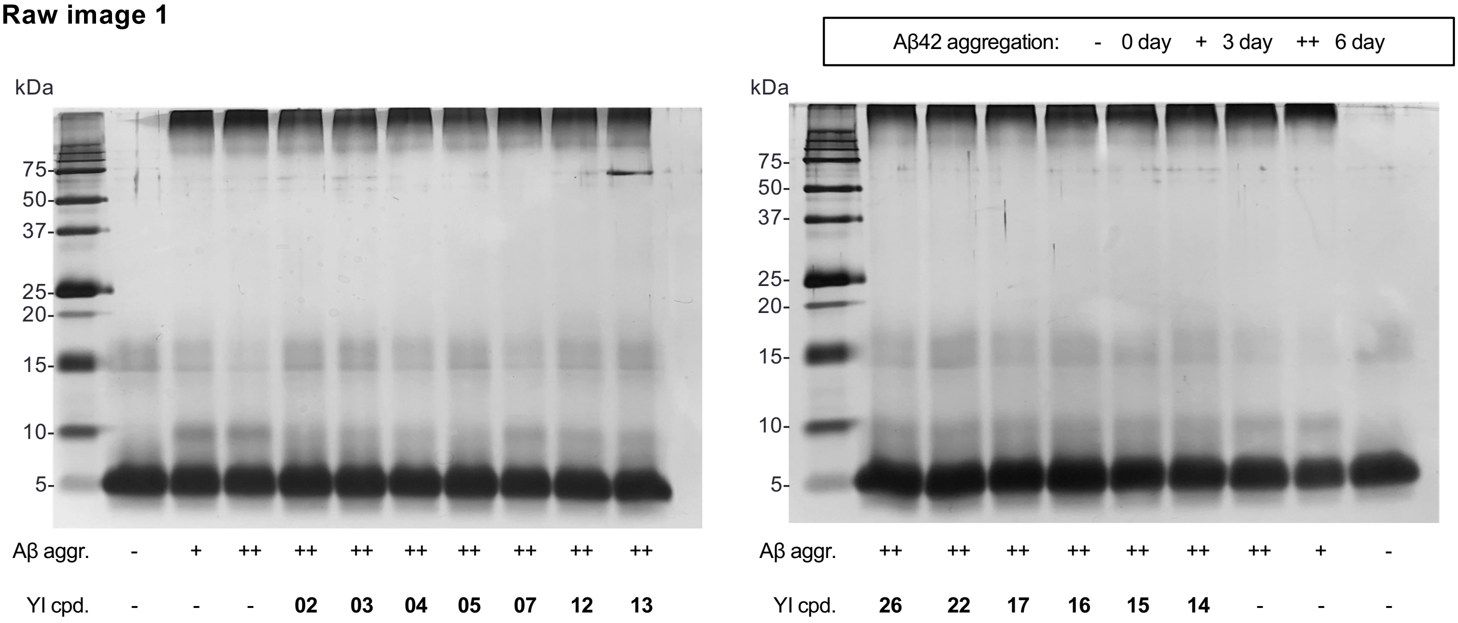

Raw image 1. Full image of SDS-PAGE analysis, related to Fig 2C. Full-length original gels of SDS-PAGE with PICUP and silver staining for disaggregation of Aβ42 (50 μM, 3-day pre-aggregation) aggregates by YI compounds (250 μM). Sizes of Aβ species according to size markers are monomers (5 kDa), dimers (10 kDa), oligomers (15 to 75 kDa), and larger aggregates or fibrils (embedded at the top of the gels). Abbreviations: – = Aβ monomer, + = 3-day incubation of Aβ, ++ = 3-day pre-incubation of Aβ and additional 3-day incubation of Aβ and/or compounds.

Raw image 2

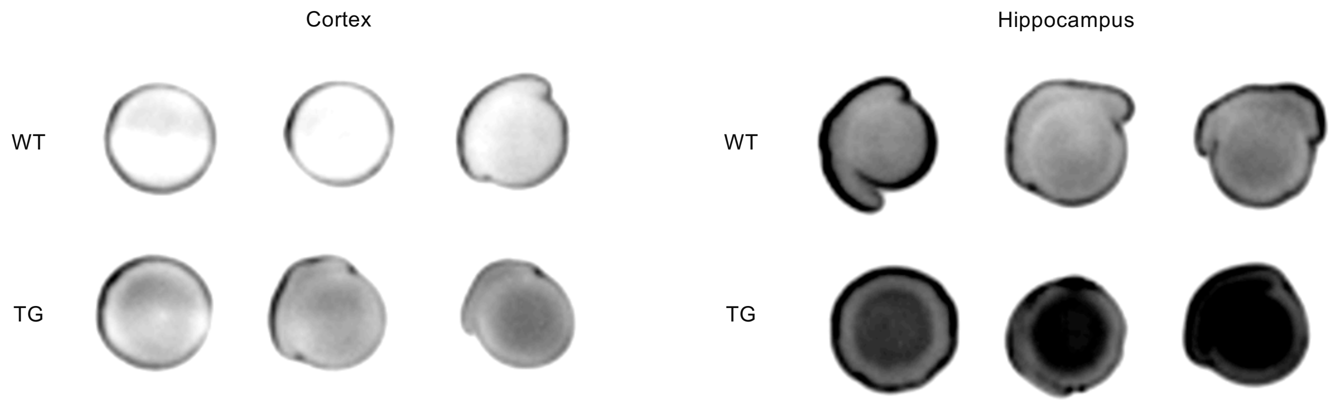

Raw image 2. Full image of dot blot assay, related to Fig 4D. Soluble Aβ oligomers were applied to a nitrocellulose membrane and probed with 6E10 which recognizes all species of Aβ. Abbreviations: WT = wild-type, TG = transgenic.
